# Modulation of Alpha and Beta Oscillations during an n-back Task with Varying Temporal Memory Load

**DOI:** 10.3389/fpsyg.2015.02031

**Published:** 2016-01-08

**Authors:** Youguo Chen, Xiting Huang

**Affiliations:** Key Laboratory of Cognition and Personality (Ministry of Education), Center of Studies for Psychology and Social Development, Faculty of Psychology, Southwest UniversityChongqing, China

**Keywords:** temporal information, duration, working memory, n-back task, neural oscillation

## Abstract

Temporal information can be retained and manipulated in working memory (WM). Neural oscillatory changes in WM were examined by varying temporal WM load. Electroencephalography was obtained from 18 subjects performing a temporal version of the visual n-back WM task (*n* = 1 or 2). Electroencephalography revealed that posterior alpha power decreased and temporal region-distributed beta power increased as WM load increased. This result is consistent with previous findings that posterior alpha band reflects inhibition of task-irrelevant information. Furthermore, findings from this study suggest that temporal region-distributed beta band activity is engaged in the active maintenance of temporal duration in WM.

## Introduction

Humans can memorize not only attributes of a presented visual stimulus but also its duration of presentation. Working memory (WM) is the system responsible for short-term storage and online manipulation of information, which is necessary for higher-order cognition, such as language, reasoning, and problem-solving (Baddeley, 1992, 2010, 2012). WM constitutes a fundamental aspect of temporal information processing, as encoded stimulus duration is temporally maintained in WM and then transferred into long-term memory. A previously encoded stimulus duration can be retrieved from long-term memory, and held in WM during a task (Gibbon, 1977; Gibbon et al., 1984; Allan, 1998; Coull et al., 2008).

Previous studies have revealed neural substrates that underlie the maintenance of stimulus duration in WM. Acetylcholine in the frontal cortex modulates the speed at which stimulus duration is translated into temporal memory representations (Meck and Church, 1987; Meck, 1996). Both working and reference memory for temporal information are sensitive to choline acetyltransferase inhibition in rats (Meck, 2006). In monkeys, stimulus duration in WM is represented by neuronal activity in prefrontal cortex (Sakurai et al., 2004). Frontal distributed alpha activity is involved in duration maintenance in WM (Chen et al., 2015). A neural network that includes the frontal lobe (left inferior frontal gyrus, right anterior cingulate, pre-supplementary motor area/supplementary motor area, right paracentral lobule, and left precentral gyrus), parietal lobe (left post-central gyrus), temporal lobe (left superior temporal gyrus), limbic system (left insula), and basal ganglia (right and left caudate and putamen) are correlated with maintenance of temporal information (Harrington et al., 2010).

Much research has addressed the maintenance of temporal duration in WM; however, few studies have investigated the manipulation of temporal duration in WM. Only one study has reported neural substrates underlying the update of temporal information in WM (Gruber et al., 2000). This study used light-emitting diodes (LEDs) that flashed with either a constant inter-stimulus interval (ISI) of 1 s or variable ISIs (0.3–1.7 s, mean = 1 s). In task 1, subjects ignored ISI changes by attempting to detect a hypothetical hidden feature of the LEDs. In task 2, subjects were required to detect ISI changes. Task 1 served as a baseline that controlled for perceptual aspects common to all tasks. During task 2, subjects were required to continually update memorized temporal information. Thus, this task was similar to a one-back WM task including perceptual processing, temporal encoding, memory updating, and comparison. More activation of prefrontal and lateral premotor cortices was observed in task 2 compared with task 1, which may engage in temporal encoding, memory updating, and comparison (Gruber et al., 2000). Typical WM tasks should be adopted to explore the maintenance and manipulation of temporal duration in WM.

The n-back task is a representative example of a WM task, because it requires manipulation as well as maintenance of information in WM (Cohen et al., 1997; Meegan et al., 2004; Owen et al., 2005). The n-back task requires participants to decide whether a currently presented stimulus matches the stimulus presented *n* trials previously. The load factor *n* can be adjusted to increase or decrease the difficulty level of the task, and to identify the neural substrates underlying WM. Various types of information can be maintained and manipulated in WM, such as letters, words, numbers, shapes, fractals, faces, pictures, locations, and auditory tones (Owen et al., 2005). Neural oscillations during *n*-back tasks have been extensively investigated (Gevins et al., 1997; McEvoy et al., 1998; Pesonen et al., 2007; Krause et al., 2010; Palomaki et al., 2012; Imperatori et al., 2013). Frontal midline theta rhythm (4–7 Hz) has been shown to increase in magnitude as memory load increases (Gevins et al., 1997, 1998; Lei and Roetting, 2011). Studies have shown that theta oscillations play an important role in WM control mechanisms (Schmiedt et al., 2005; Sauseng et al., 2009). In particular, theta oscillations reflect the organization of sequentially ordered items in WM (Hsieh et al., 2011; Roberts et al., 2013; Roux and Uhlhaas, 2014). In contrast, posterior alpha band power (7.5–12 Hz) has been shown to decrease as memory load increases (Gevins et al., 1997, 1998; Lei and Roetting, 2011). Alpha oscillations tend to be attenuated by attention-demanding tasks, reflecting the inhibition of cortical areas that represent task-irrelevant information (Gevins and Smith, 2000; Jokisch and Jensen, 2007; Klimesch et al., 2007; Tuladhar et al., 2007; Manza et al., 2014). The role of the beta band (13–35 Hz) in WM remains under debate. One study found that beta band frequency increases over the parietal region as memory load increases (Deiber et al., 2007). The authors of this study proposed that the beta band is related to item retention and active maintenance for further task requirements. In contrast, other studies have reported that increased WM load is associated with beta desynchronization (i.e., decrease in beta power; Bocková et al., 2007; Pesonen et al., 2007; Krause et al., 2010). It has been proposed that beta oscillations correlate with higher WM performance due to more effective filtering of irrelevant information (Zanto and Gazzaley, 2009).

The present study applied an n-back task to investigate neural oscillations that underlie manipulation and maintenance of temporal duration in WM. Neural substrates that underlie an increase in temporal WM load can be identified by parametric changes in *n*. In a temporal version of the n-back task, the participant is shown a series of items (e.g., red circles) and asked to decide whether the duration of presentation of the current item matches the duration of the item presented *n* trials back. The task requires manipulation and maintenance of temporal information in WM. As stated previously, theta and alpha bands reflect central executive functions of WM (Sauseng et al., 2005). Specifically, theta band oscillations reflect the organization of sequentially ordered WM items (Schmiedt et al., 2005; Sauseng et al., 2009; Hsieh et al., 2011; Roberts et al., 2013; Roux and Uhlhaas, 2014) and alpha oscillations reflect inhibition of task-irrelevant information (Gevins and Smith, 2000; Jokisch and Jensen, 2007; Klimesch et al., 2007; Tuladhar et al., 2007; Manza et al., 2014). According to the “multiple-component model” by Baddeley and Hitch, unique central executive control mechanisms, such as item organization and inhibition of irrelevant information (Bledowski et al., 2010), are activated for different types of information in WM (Baddeley, 1992, 2010, 2012). We hypothesized that frontal theta would increase and posterior alpha would decrease as temporal WM load increased. As previously stated, the role of the beta band in WM remains under debate. If beta oscillations are related to the maintenance of item information (Deiber et al., 2007), then we would expect beta band power to increase as temporal WM load increases. In contrast, if beta oscillations are like alpha oscillations, which have been associated with inhibition of task-irrelevant information (Zanto and Gazzaley, 2009; Waldhauser et al., 2012), then we would expect to observe a decrease in beta band power (beta desynchronization) as temporal WM load increases.

## Materials and Methods

### Participants

Eighteen right-handed undergraduate students (eight male students, 19–24 years of age) were paid for their participation in this experiment. Each participant had normal or corrected-to-normal visual acuity. Participants were not taking any medications and did not suffer from any central nervous system abnormalities or injuries. The study was approved by the local institutional review board. Written informed consent was obtained from each participant. The experimental procedure was conducted in accordance with the Declaration of Helsinki (World Medical Association, 2013).

### Experimental Material and Apparatus

Visual stimuli were displayed on a black background in the center of a computer screen. A 3-cm red circle (2.29°) and a white 2-cm question mark (1.53°) were used as visual stimuli. Four presentation durations were chosen for the red circle. Scalar variability, in which the standard deviation of the estimated intervals increases linearly with their mean, is a verified feature associated with temporal processing (Rakitin et al., 1998; Brannon et al., 2008). Thus, an exponential function was adopted to select durations to match the difficulty of discrimination between each pair of adjacent durations. The four durations were: 100 (100 × 2^0^), 200 (100 × 2^1^), 400 (100 × 2^2^), and 800 (100 × 2^3^) ms. The refresh rate of the computer monitor was 85 Hz, and the computer screen was placed approximately 75 cm from the participant during the task.

### Procedure

The temporal version of the n-back task was used in this study. A 1-back task was defined as low load (LL), and a 2-back task was defined as high load (HL). The order of the two memory load conditions was counterbalanced across subjects. There were four blocks for each memory load condition, and 25 trials for each duration in each block.

**FIGURE 1:**
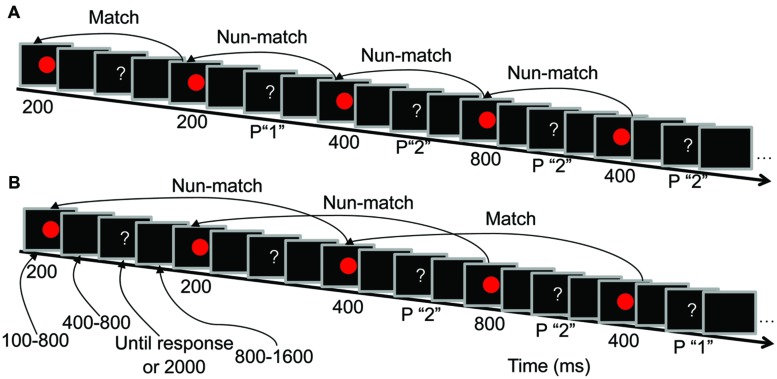
**Trial sequences and the duration of each screen presentation. (A)** Low load condition (LL, 1-back task) procedures. **(B)** High load condition (HL, 2-back task) procedures. P “1” indicates that pressing “1” is the correct response, and P “2” indicates that pressing “2” is the correct response.

The trial sequence was identical for the 1-back and 2-back tasks (**Figure 1**). Temporal jitter between stimuli was used (Luck, 2005) to reduce the distortion that results from overlapping neural activity between previous and subsequent stimuli. Randomized temporal jitter was controlled by E-prime 1.1 (Psychology Software Tools, Inc.). During each trial, a red circle was presented for a randomly selected duration (100, 200, 400, or 800 ms). After a random delay of 400–800 ms, a question mark was presented in the center of the screen until a response was made, or for a maximum of 2000 ms. Participants were informed that they had to respond within 2000 ms. Trials were presented with a random inter-trial interval of 800–1600 ms.

Participants performed a duration comparison task in which they were required to remember the presentation duration of the red circle at two levels of difficulty (LL and HL). In the LL condition, participants indicated whether the duration of the current red circle was the same as that of the previous red circle (**Figure 1A**). In the HL condition, participants indicated whether the duration of the current red circle was the same as that of the red circle presented two presentations previously (**Figure 1B**). The percentages of matched and unmatched trials were both 50% in both the 1-back and 2-back tasks. When the question mark was presented, participants were instructed to press “1” if the memorized durations of the two red circles were the same and “2” if the memorized durations were different. Half of the participants responded with their left hand (pressing “1” with their middle finger and “2” with their index finger), and the other half responded with their right hand (pressing “1” with their index finger and “2” with their middle finger).

### Electrophysiological Recording

Continuous electroencephalography (EEG) was acquired from Ag/AgCl electrodes mounted in an elastic cap (Brain Products GmbH, Gilching, Germany). Sixty-four electrodes were positioned according to the extended 10–20 system. Additional electrodes were placed on the mastoids. Horizontal electrooculograms (EOGs) were acquired using bipolar electrodes positioned at the external ocular canthi, and vertical EOGs were recorded from electrodes placed above and below the left eye. The EEG and EOG were digitized at 500 Hz with an amplifier bandpass of 0.01–100 Hz, including a 50-Hz notch filter, and stored for oﬄine analysis. All electrode impedances were maintained below 5 kΩ.

### EEG Analysis

EEGLAB (Delorme and Makeig, 2004) and MATLAB (The MathWorks, Natick, MA, USA) were used for oﬄine EEG data processing. Continuous EEG data were re-referenced to the average of the right and left mastoids, and digitally low-pass filtered at 40 Hz. EEG epochs were segmented in 3-s time windows (pre-stimulus 1 s and post-stimulus 2 s, 0 was onset of stimulus) and baseline-subtracted in the time domain from –1000 to 0 ms. Baseline correction in the time domain effectively subtracts the direct current with no impact on frequency components (Addante et al., 2011). Trials with EOG artifacts (mean EOG voltage exceeding ± 80 μV) and those contaminated with artifacts due to amplifier clipping or peak-to-peak deflection exceeding ±80 μV were excluded. Remaining EOG artifacts were visually identified and removed using independent component analysis according to scalp maps and activity profiles; independent components related to eye movements had a large EOG channel contribution and a frontal scalp distribution (Jung et al., 2000a,b).

Segmented and artifact-free data were used for power spectral analysis. Time-frequency EEG power data were obtained using Hanning-windowed sinusoidal wavelets of three cycles at 3 Hz, rising linearly to approximately 20 cycles at 40 Hz (Gevins et al., 1997; Makeig et al., 2004). The present study focused on ongoing EEG power rather than event-related changes in the power spectrum (Gevins et al., 1997, 1998; Gevins and Smith, 2000; Lei and Roetting, 2011). Thus, the pre-stimulus baseline was not subtracted from ongoing EEG power (Addante et al., 2011; **Figure 4**).

Following previous studies (Hsieh et al., 2011; Chen et al., 2015), electrodes were grouped into nine different clusters: left-frontal (AF7, F7, F5), middle-frontal (F1, Fz, F2), right-frontal (AF8, F8, F6), left-central (C3, C5, T7), middle-central (C1, Cz, C2), right-central (C4, C6, T8), left-posterior (P5, P7, PO7), middle-posterior (O1, O2, Oz), and right-posterior (P6, P8, PO8).

Theta band (4–7 Hz), alpha band (7.5–12 Hz), and beta band (13–34 Hz) powers were analyzed separately. These oscillatory bands were defined by the conventional International Federation of Clinical Neurophysiology (IFCN) guidelines (Nuwer et al., 1999). As shown in **Figure 4**, a posterior alpha decrease and a temporal region-distributed beta increase were observed with increasing WM load from –400 to 1400 ms. Three-way repeated-measures analyses of variance (ANOVAs) were conducted on mean theta, alpha, and beta power in the –400 to 1400 ms time intervals with factors memory load (LL and HL), duration (100, 200, 400, and 800 ms) and region (nine electrode clusters). A Greenhouse–Geisser correction was used to correct for any violations of sphericity (Greenhouse and Geisser, 1959).

## Results

### Behavioral Data

**FIGURE 2:**
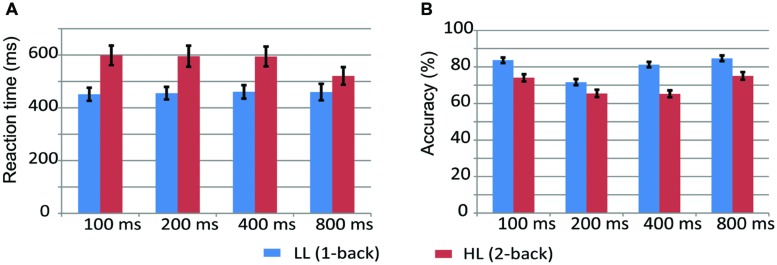
**Reaction time and accuracy in the LL (A, 1-back) and HL (B, 2-back) conditions**.

**Figure 2** displays the mean values and standard error of accuracy and reaction time (RT) for 100-, 200-, 400-, and 800-ms durations in the LL and HL conditions. A two-way repeated measures ANOVA on RT with memory load and duration as within-participant factors revealed significant main effects of memory load [*F*(1,17) = 21.966, *p* < 0.001, $\eta_{p}^{2}$ = 0.564] and duration [*F*(3,51) = 7.086, *p* < 0.01, $\eta_{p}^{2}$ = 0.294], and a memory load × duration interaction [*F*(1.971,33.511) = 9.567, *p* < 0.01, $\eta_{p}^{2}$ = 0.360]. Simple effects analyses on the memory load × duration interaction revealed that RT was longer in the HL condition than the LL condition for all durations [100 ms: *F*(1,17) = 31.570, *p* < 0.001, $\eta_{p}^{2}$ = 0.650; 200 ms: *F*(1,17) = 22.136, *p* < 0.001, $\eta_{p}^{2}$ = 0.566; 400 ms: *F*(1,17) = 24.098, *p* < 0.001, $\eta_{p}^{2}$ = 0.586; 800 ms: *F*(1,17) = 4.466, *p* = 0.05, $\eta_{p}^{2}$ = 0.280].

A repeated-measures ANOVA on accuracy revealed significant main effects of memory load [*F*(1,17) = 51.169, *p* < 0.001, $\eta_{p}^{2}$ = 0.751] and duration [*F*(3,51) = 33.503, *p* < 0.001, $\eta_{p}^{2}$ = 0.663], and a significant memory load × duration interaction [*F*(2.239,38.04) = 11.484, *p* < 0.001, $\eta_{p}^{2}$ = 0.403]. Simple effects analyses on the memory load × duration interaction revealed that accuracy was lower in the HL condition than the LL condition for all durations [100 ms: *F*(1,17) = 34.713, *p* < 0.001, $\eta_{p}^{2}$ = 0.671; 200 ms: *F*(1,17) = 13.523, *p* < 0.01, $\eta_{p}^{2}$ = 0.443; 400 ms: *F*(1,17) = 114.192, *p* < 0.001, $\eta_{p}^{2}$ = 0.870; 800 ms: *F*(1,17) = 18.280, *p* < 0.01, $\eta_{p}^{2}$ = 0.518].

**FIGURE 3:**
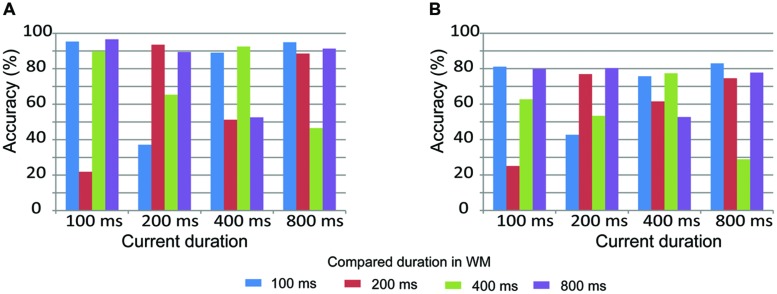
**Pairwise comparisons of duration accuracy in the LL (A, 1-back task) and HL (B, 2-back task) conditions**.

Pairwise comparisons of duration accuracy are displayed in **Figure 3** to further understand how participants compare the current duration with the duration stored in WM. Similar results were observed on both the 1-back and 2-back tasks. Accuracy was low when the current duration was adjacent to the compared duration. For example, accuracy was low when a current duration of 200 ms was compared to adjacent durations of 400 or 600 ms. In contrast, accuracy was high when the current duration was equal to or not adjacent to the compared duration. For example, accuracy was high when a current duration of 200 ms was compared to an equal duration of 200 ms or a non-adjacent duration of 800 ms. These results suggest that durations were effectively maintained in WM in both the 1-back and 2-back tasks.

### EEG Data

**FIGURE 4:**
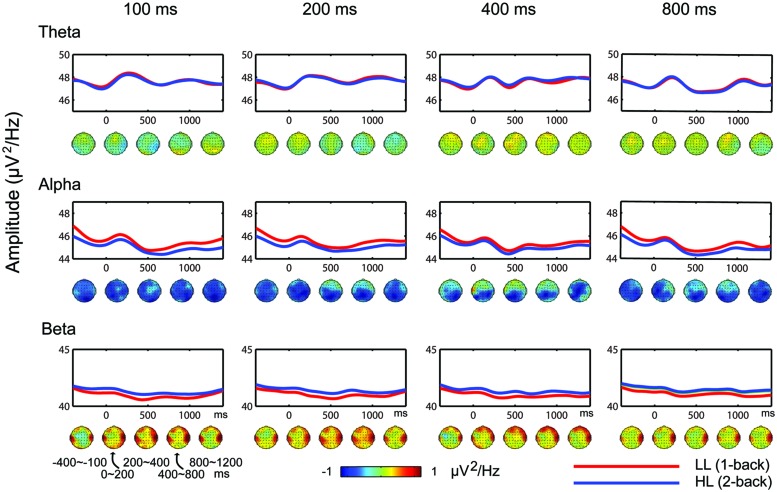
**Temporal dynamic activity of theta, alpha, and beta oscillations for 100-, 200-, 400-, and 800-ms duration conditions in the time interval from –400 to 1400 ms**. Red and blue lines indicate the mean spectral power of nine clusters. The topographies indicate the distributions of the HL minus LL power difference during the time intervals of –400 to –100, 0 to 200, 200 to 400, 400 to 800, and 800 to 1200 ms.

Similar results were obtained from the 100-, 200-, 400-, and 800-ms durations (**Figure 4**). Theta band oscillations (4–7 Hz) were similar between the HL and LL conditions. Alpha band power (7.5–12 Hz) over the posterior region from –400 to 1400 ms was lower in the HL condition than the LL condition. Beta band power (13–35 Hz) over the temporal region from –400 to 1400 ms was higher in the HL condition than the LL condition. These results are consistent with the higher WM load that participants are under in the 2-back task, even during the inter-trial interval.

**FIGURE 5:**
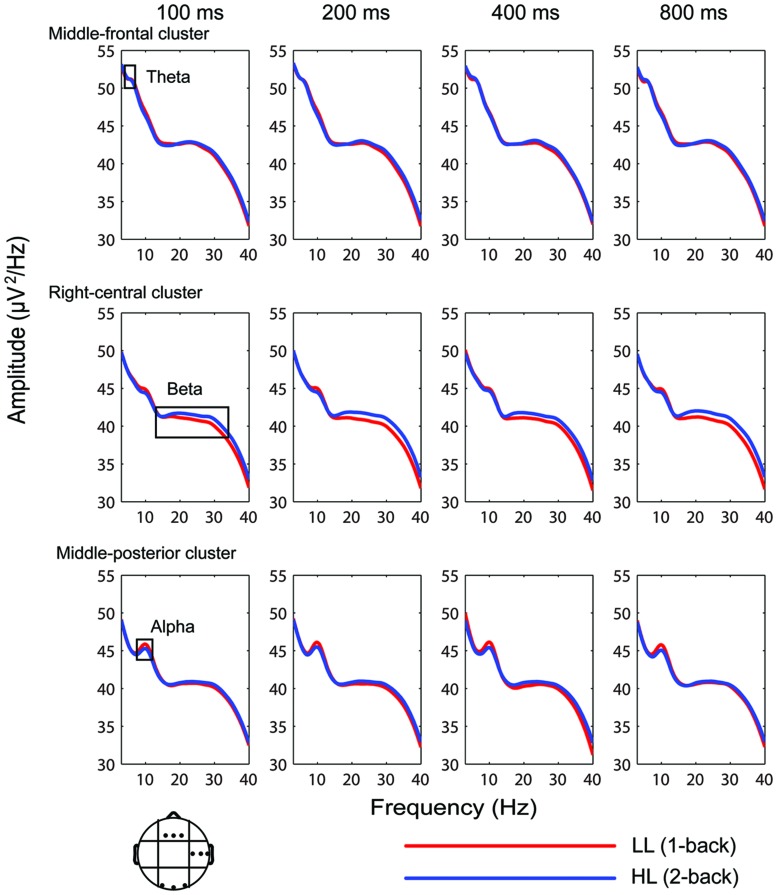
**Average EEG spectral power (–400 to 1400 ms) for the 100-, 200-, 400-, and 800-ms durations under the low (LL) and high (HL) memory load conditions in the middle-frontal, right-central, and middle-posterior clusters**.

Electroencephalography spectral power was averaged over the time interval from –400 to 1400 ms (**Figure 5**). ANOVA conducted on theta band power (4–7 Hz) revealed significant main effects of duration [*F*(2.506,42.608) = 20.262, *p* > 0.001, $\eta_{p}^{2}$ = 0.544] and region [*F*(3.648,62.013) = 108.840, *p* < 0.001, $\eta_{p}^{2}$ = 0.865]. Theta band amplitude was significantly lower in the 800-ms condition compared with the 100, 200, and 400-ms conditions (*p*-values < 0.001); the differences between the 100, 200, and 400-ms conditions were not significant (*p*-values > 0.05). Theta power was highest over the middle-frontal cluster (51.056 ± 0.441 μV^2^/Hz). Main effects of memory load and interactions of memory load × duration, memory load × region, duration × region, and memory load × duration × region were not significant (*p*-values > 0.05).

Analysis of variance conducted on alpha band power revealed significant effects of memory load [*F*(1,17) = 12.945, *p* < 0.01, $\eta_{p}^{2}$ = 0.432], duration [*F*(2.054,34.912) = 4.830, *p* < 0.05, $\eta_{p}^{2}$ = 0.221], region [*F*(2.525,42.918) = 16.270, *p* < 0.001, $\eta_{p}^{2}$ = 0.489], and duration × region interaction [*F*(6.773,115.145) = 3.045, *p* < 0.001, $\eta_{p}^{2}$ = 0.152]. Alpha band power was higher in the LL condition (45.247 ± 0.759 μV^2^/Hz) than the HL condition (44.810 ± 0.704 μV^2^/Hz). Simple effects analyses on the duration × region interaction revealed a significant effect of duration over the left-frontal [*F*(3,15) = 3.644, *p* < 0.05, $\eta_{p}^{2}$ = 0.422], middle-central [*F*(3,15) = 3.880, *p* < 0.05, $\eta_{p}^{2}$ = 0.437], left-posterior [*F*(3,15) = 3.977, *p* < 0.05, $\eta_{p}^{2}$ = 0.443], and middle-posterior [*F*(3,15) = 10.526, *p* < 0.01, $\eta_{p}^{2}$ = 0.678] clusters such that the alpha power band amplitude was significantly lower in the 800-ms condition than the 100, 200, and 400-ms conditions. Interactions of memory load × duration, memory load × region, and memory load × duration × region were not significant (*p*-values > 0.05).

Analysis of variance conducted on beta band power revealed significant effects of memory load [*F*(1,17) = 6.439, *p* < 0.05, $\eta_{p}^{2}$ = 0.275], region [*F*(2.728,46.378) = 15.112, *p* < 0.001, $\eta_{p}^{2}$ = 0.471], and memory load × region interaction [*F*(3.586,60.969) = 3.064, *p* < 0.05, $\eta_{p}^{2}$ = 0.153]. Simple effects analyses on the memory load × region interaction revealed that beta band power was significantly lower in the LL condition than the HL condition over the right-frontal [*F*(1,17) = 4.760, *p* < 0.05, $\eta_{p}^{2}$ = 0.219], left-central [*F*(1,17) = 7.890, *p* < 0.05, $\eta_{p}^{2}$ = 0.317], and right-central [*F*(1,17) = 13.262, *p* < 0.01, $\eta_{p}^{2}$ = 0.438] clusters. The main effect of duration and memory load × duration, duration × region, and memory load × duration × region interactions were not significant (*p*-values > 0.05).

**FIGURE 6:**
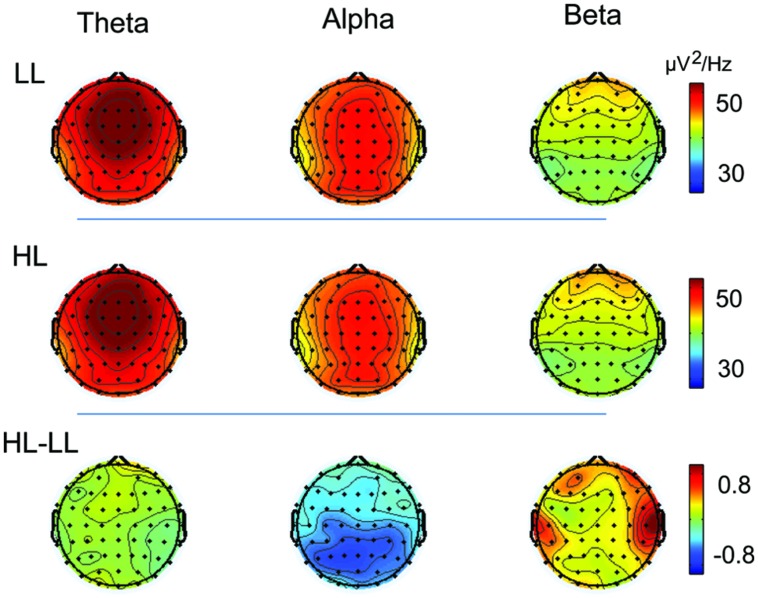
**The topographies of theta, alpha, and beta activity in the LL and HL conditions, and the HL minus LL power difference**.

Given that similar results were obtained across all four duration conditions (**Figures 4** and **5**), the oscillation power was averaged across durations to plot the topographies of the oscillations. Theta band was highest over the frontal region, alpha band was highest over the frontal, central, and parietal regions, and beta band was highest over the frontal region in both the LL and HL conditions. LL subtracted from HL revealed an alpha decrease distributed over the posterior region and a beta increase distributed over the temporal region (**Figure 6**).

## Discussion

Accuracy decreases and RT increases with increasing WM load on spatial and verbal versions of the n-back task (Gevins et al., 1997; McEvoy et al., 1998). The present study found that accuracy was decreased and RT was increased in the HL condition (2-back task) compared with the LL condition (1-back task) for the 100-, 200-, 400-, and 800-ms duration conditions (**Figure 2**), which suggests that memory load was effectively manipulated. We found a significant memory load × duration interaction on RT. This significance was driven by a smaller difference in RT between the 1-back and 2-back task in the 800-ms condition [mean difference (MD): 61.06 ms] compared with the 100-ms (MD: 147.54 ms), 200-ms (MD: 140.07 ms), and 400-ms (MD: 133.85 ms) conditions. Similarly, a significant memory load × duration interaction on accuracy is due to a larger difference in accuracy between the 1-back and 2-back tasks in the 400-ms condition (MD: 16.0%) compared with the 100-ms (MD: 9.6%), 200-ms (MD: 6.2%), and 800-ms (MD: 9.6%) conditions. These interactions did not influence the effective manipulation of memory load, and therefore, they will not be further discussed.

Time-frequency analysis was conducted on EEG data to identify the temporal dynamic activity of oscillations (**Figure 4**). Decreases in alpha band and increases in beta band were observed with increasing temporal WM load from –400 to 1400 ms. This result suggests that the WM load is higher in the 2-back task than the 1-back task even during the inter-trial interval. For this reason, the present study focused on ongoing EEG power rather than event-related changes in the power spectrum. If the pre-stimulus baseline is subtracted from ongoing EEG power, then neural activity related to WM load would be removed. Furthermore, alpha band decreases and beta band increases emerged during the time interval from –400 to 0 ms (**Figure 4**), a phase during which temporal encoding (i.e., timing) does not exist. Thus, this result indicates that alpha band decreases and beta band increases are due to increased WM load rather than temporal encoding. In addition, the present study found that theta and alpha band amplitudes were lower in the 800-ms condition than the 100-, 200-, and 400-ms conditions. This result may represent neural oscillatory correlates of temporal encoding, and will not be further discussed.

Consistent with previous studies on WM (Gevins et al., 1997, 1998; Gevins and Smith, 2000; Jensen and Tesche, 2002; Onton et al., 2005; Lei and Roetting, 2011), a pronounced theta power was distributed over the frontal midline in both the LL and HL conditions (**Figure 6**). This theta activity emanates from the anterior cingulate cortex (Onton et al., 2005; Womelsdorf et al., 2010). The present study found that theta power was not modulated by increasing temporal memory load. To determine whether this result was due to the time-frequency analysis method, time-frequency EEG power data were obtained using Hanning-windowed sinusoidal wavelets of three cycles at 3 Hz (Makeig et al., 2004). This analysis method was previously adopted to extract frontal midline theta during a Sternberg WM task (Onton et al., 2005). We performed a supplementary analysis in which each set of EEG data (5-s epoch) was subjected to Fast-Fourier Transform (FFT) analysis (Chen et al., 2008). No distinct difference in theta band power between the LL and HL conditions was observed. This result suggests that the lack of an effect of temporal WM load on theta band power is not due to the time-frequency analysis method.

Previous studies have shown that theta band reflects the organization of sequentially ordered items in WM (Hsieh et al., 2011; Roberts et al., 2013; Roux and Uhlhaas, 2014). The number of temporal order relationships among items in WM increases as WM load increases, which in turn increases the amplitude of theta band power (Hsieh et al., 2011). This finding was not confirmed in the present study. In previous studies, letters, digits, locations, or visual objects were held in WM, and the visual representation of each item was different (Roland and Gulyas, 1994). In the present study, one duration was stored in WM for the 1-back task, and two durations were stored in WM for the 2-back task. However, the same red circle was presented in each trial, and thus the visual representation of each item was identical in the LL and HL conditions. Our results suggest that the amplitude of the theta band increases as a function of the number of temporal order relationships only when different visual representations are stored in WM. This hypothesis should be tested further in future studies.

Consistent with previous n-back studies (Gevins et al., 1997, 1998; Gevins and Smith, 2000; Lei and Roetting, 2011), the present study found that alpha power decreased with increased memory load. This result is consistent with the finding that increases in alpha oscillation amplitudes reflect increases in cortical inhibition, and decreases in alpha band reflect task-relevant cortical activity (Pfurtscheller, 2001; Klimesch et al., 2007). Functional neuroimaging studies revealed that areas involved in WM (prefrontal and parietal cortex) vary as a function of memory load, with greater activation for higher load levels (Cohen et al., 1997; Owen et al., 2005). Thus, decreases in alpha band in posterior sites reflect increases in cortical activity with increased memory load.

The present study supports the role of beta band oscillations in maintenance rather than inhibition. Given that alpha and beta oscillations are proposed to reflect inhibition of interfering visual memories (Waldhauser et al., 2012), decreased beta band would be expected to be observed with an increased WM load (Zanto and Gazzaley, 2009). However, others have proposed that beta oscillations are related to the maintenance of item information, such that beta band power would increase with increased temporal WM load (Deiber et al., 2007). The beta band increase in the present study supports the maintenance hypothesis. This result is consistent with previous studies that beta increase is associated with maintaining an existing steady state in motor control (Gilbertson et al., 2005; Pogosyan et al., 2009). The present study found that the increased beta was largest over temporal region, which is in agreement with a neuroimaging study that cortico-striatal circuits and superior temporal lobe engage in maintenance of duration in WM (Harrington et al., 2010). Previous studies revealed that phase synchrony in beta oscillations plays an important role for connectivity and communication between/within cortico-striatal circuits and auditory cortex (Fujioka et al., 2012), which may explain how beta oscillations maintain information in neaural networks.

Our research will inspire future studies on temporal information processing. First, as the first step, we showed that n-back task is suitable for studying maintenance and manipulation of duration in WM, and revealed functions of alpha and beta bands in maintenance and manipulation of duration in WM. This experimental paradigm can be used to identify several unsolved scientific problems about representation of duration in WM. E.g., Whether auditory and visual duration is represented differently in WM; whether there is any difference in representations between short and long durations. Second, comparing with previous studies, our study revealed a specific neural activity pattern for duration maintenance in WM. We found that temporal region-distributed beta bands reflect maintenance of duration in WM. Deiber et al. (2007) demonstrated the reactivity of the beta oscillations to the verbal WM load, more pronounced in the right parietal region. Differences in topographies of beta bands are consistent with a previous meta-analysis study which revealed subregional and lateralized differences in activation of a frontoparietal network in response to contents of WM (such as locations, letters, sharps; Owen et al., 2005). Our study indicates the specific neural activity pattern for temporal WM load which can be further identified using n-back task combining with magnetoencephalogram (MEG) or functional magnetic resonance imaging (fMRI). Third, our study proposed an open question. It is not clear why isn’t theta power modulated by increasing temporal memory load. It indicates that there are certain differences in organization of durations and other types of information (such as locations, letters). By solving this open question, it is helpful to understand how temporal durations are organized in WM.

To summarize, the present study applied an n-back paradigm to explore neural oscillatory correlates of maintenance and manipulation of duration in WM. We found that frontal midline theta activity was not modulated by increased duration memory load, whereas alpha power was decreased over the posterior region and beta power was increased over the temporal region in the HL compared with the LL condition. The relationship between theta band and the organization of duration in WM needs to be further investigated. Our results are consistent with previous studies in which posterior alpha band was shown to reflect the inhibition of task-irrelevant information. This study also revealed an important role of temporal region-distributed beta in the active maintenance of duration in WM.

## Author Contributions

YC and XH developed the experimental concept and the design. YC collected data. YC performed the data analysis and interpretation under the supervision of XH. YC and XH wrote the manuscript. All authors approved the final version of the manuscript for submission.

## Conflict of Interest Statement

The authors declare that the research was conducted in the absence of any commercial or financial relationships that could be construed as a potential conflict of interest.
